# Hamstring-to-quadriceps ratio across menstrual cycle phases in physically active women: a repeated-measures study

**DOI:** 10.1007/s00421-026-06279-6

**Published:** 2026-06-02

**Authors:** Christine Heinrich, Hanna Hofstaetter, Sebastian Puschkasch-Möck, Michael Behringer

**Affiliations:** 1 Department of Sports Sciences, Goethe University Frankfurt, Frankfurt/Main, Germany; 2 Olympic Training and Testing Center of Hessen, Frankfurt/Main, Germany

**Keywords:** Hamstring-to-quadriceps ratio, Female athlete, Injury risk, Menstrual cycle

## Abstract

**Objective:**

To evaluate conventional hamstring-to-quadriceps ratio across the menstrual cycle in distinct angular velocities in an eumenorrheic, physically active population.

**Design:**

Repeated measures within-subjects design.

**Methods:**

Assessments were conducted in early follicular, ovulatory, and mid-luteal phase in *n* = 30 eumenorrheic females (age 27.2 yrs ± 4.1; height 168.3 cm ± 5.6; body mass 64.7 kg ± 7.5; body fat 25.9% ± 4.4; fat free mass 47.0 kg ± 4.7, training hours 6.98 ± 3.01, cycle length 28.2 ± 2.9). Isokinetic concentric peak torque values of leg extension and leg flexion at 60°∙s^− 1^ and 120°∙s^− 1^ angular velocity were recorded. Participants performed five consecutive maximum repetitions, and the highest values were obtained for further analysis. Subsequently, conventional hamstring-to-quadriceps peak torque ratio (HQ Ratio) was calculated by dividing the highest peak torque value of leg flexion by the highest peak torque value of leg extension.

**Results:**

HQ Ratio did not differ significantly across menstrual cycle phases at 60°∙s^− 1^ (F (2,51) = 0.86, *p* = 0.43), or at 120°∙s^− 1^ (F (2, 52) = 0.46, *p* = 0.64). Peak torque values of leg flexion did not differ significantly between menstrual cycle phases at 60°∙s^− 1^ (F (2,50 = 2.43, *p* = 0.1) or at 120°∙s^− 1^ (F (2,52) = 0.48, *p* = 0.62). Furthermore, peak torque values of leg extension remained unaltered across distinct menstrual cycle phases at 60°∙s^− 1^ (F (2,50) = 0.5, *p* = 0.58), and at 120°∙s^− 1^ (F (2,51) = 0.29, *p* = 0.75).

**Conclusion:**

Strength ratios of leg extensors and flexors do not seem to differ between cycle phases. Isokinetic peak torque values of knee extension and flexions seem to remain constant across distinct menstrual cycle phases. Results indicated that conventional isokinetic peak torque testing does not need to be scheduled according to the menstrual cycle.

## Background

In recent years, the professionalization of women’s sports has gained momentum; and with a higher participation in sports, a higher number of injuries has been recorded among recreational and elite athletes (Mallorquín et al. 2025). While the total number of treated injuries differs between men and women, their injury profiles also vary. As highlighted in both media and scientific literature, the incidence of anterior cruciate ligament ruptures (ACLR) is considerably higher in women, who are reported to have a 2–6 times greater risk compared to men (Bruder et al. 2023). Furthermore, the probability of women returning to sports or returning to competitive sports after an ACL tear is 1.5 to 1.7 times lower than in male counterparts (Ardern et al. 2014).

Reasons for this gender disparity appear to be multifactorial: Biomechanical, anatomical, and neuromuscular characteristics are discussed as possible risk factors. Women tend to have an increased Q-angle and pelvis width, while femoral notch width is smaller, leading to an increased valgus and an unfavorable force vector (Hewett et al. 2006). Furthermore, strength imbalances between leg extensors and flexors could be a further risk factor for injuries (Möck et al. 2023). Hamstring flexibility may be increased, while often underrecruited or weak, resulting in a disadvantageous antagonist-agonist-relationship that limit co-contraction of hamstring muscles to protect ligaments (Hewett et al. 2006).

Besides anatomical and biomechanical reasons, hormonal influences on ligaments and skeletal muscle tissues are further discussed as probable rationale. It has been shown that fluctuations in steroid hormones alter stiffness of ligaments, leading to increased knee laxity when hormone concentrations are elevated (Yu et al. 1999, Legerlotz and Nobis 2022). Consequently, knee laxity seems to vary across the menstrual cycle and is highest during ovulatory phase, when estradiol concentrations peak (Somerson et al. 2019, Herzberg et al. 2017). Regarding skeletal muscle tissues, Bell et al. (2011) reported that hamstring extensibility was higher at ovulation compared to menstruation, supporting an influence of steroid hormones on muscle tissue stiffness (Bell et al. 2011).

Furthermore, neuromuscular control and recruitment may be altered throughout the menstrual cycle. Khowailed et al. (2015) measured neuromuscular control patterns using electromyography in twelve females and reported a decrease in hamstring activity during ovulation when estradiol concentrations are at their highest. They further demonstrated that hamstring-to-quadriceps co-contraction ratio decreased during early follicular phase, leading to the assumption that activation patterns change throughout the cycle. However, since assessments were only conducted during menses and at ovulation, evidence remains inconclusive regarding other cycle phases. Pournasiri et al. (2023) investigated HQ ratio at 60°∙s^− 1^ angular velocity during three phases of the menstrual cycle and showed changes in maximum strength of flexors and extensors; however, HQ ratio remained stable across the cycle (Pournasiri et al. 2023).

As shown, there is only insufficient research regarding the ratio between hamstring and quadriceps strength across the menstrual cycle. Furthermore, the research available is inconclusive since only distinct menstrual cycle phases or angular velocities were represented. Hence, the aim of this study was to complement existing evidence by investigating ratios of maximal peak torque of leg flexors and leg extensors in early follicular, ovulatory, and mid-luteal phase at multiple angular velocities.

We hypothesized that HQ ratios would vary across menstrual cycle phases due to potential changes in hamstring recruitment associated with alterations in neuromuscular activation patterns. A clearer understanding of potential fluctuations in muscle strength and strength ratios of leg extensors and flexors across the menstrual cycle may contribute to more informed training, rehabilitation, and injury-prevention strategies for female athletes.

## Methods

### Study design

The assessment was embedded in a larger study conducted at Goethe University Frankfurt between September 2023 and March 2025. The study followed an observational design over one menstrual cycle, with each participant being assessed during the early follicular, ovulatory, and mid-luteal phase. All participants were measured in a fixed sequence, beginning in the early follicular phase and ending in the mid-luteal phase, due to feasibility constraints.

Ethical approvement was given by the local ethics committee and preregistered at German Clinical Trial Register (trial number: DRKS00032152). All evaluations have been conducted in accordance with the Declaration of Helsinki.

### Participants

Criteria for eligibility were (1) eumenorrheic menstrual cycle between 21 and 35 days, (2) no prior ACL tear, (3) absence of acute injuries of the lower extremities, (4) physically active, categorized at least as Tier 1 athletes (McKay et al. 2021).

Thirty eumenorrheic females (age 27.2 yrs ± 4.1; height 168.3 cm ± 5.6; body mass 64.7 kg ± 7.5; body fat 25.9% ± 4.4; fat free mass 47.0 kg ± 4.7) were recruited. A rise of luteinizing hormone in urine was observed in all participants mid-cycle, indicating ovulation. The participants could be classified as physically active with 6.98 (± 3.01) training hours per week. Their athletic background was diverse and compromised following sports: running, strength training, tennis, soccer, volleyball, badminton, rhythmic sport gymnastics, combat sports, dancing, Olympic weightlifting.

### Menstrual cycle verification

**Fig. 1:**
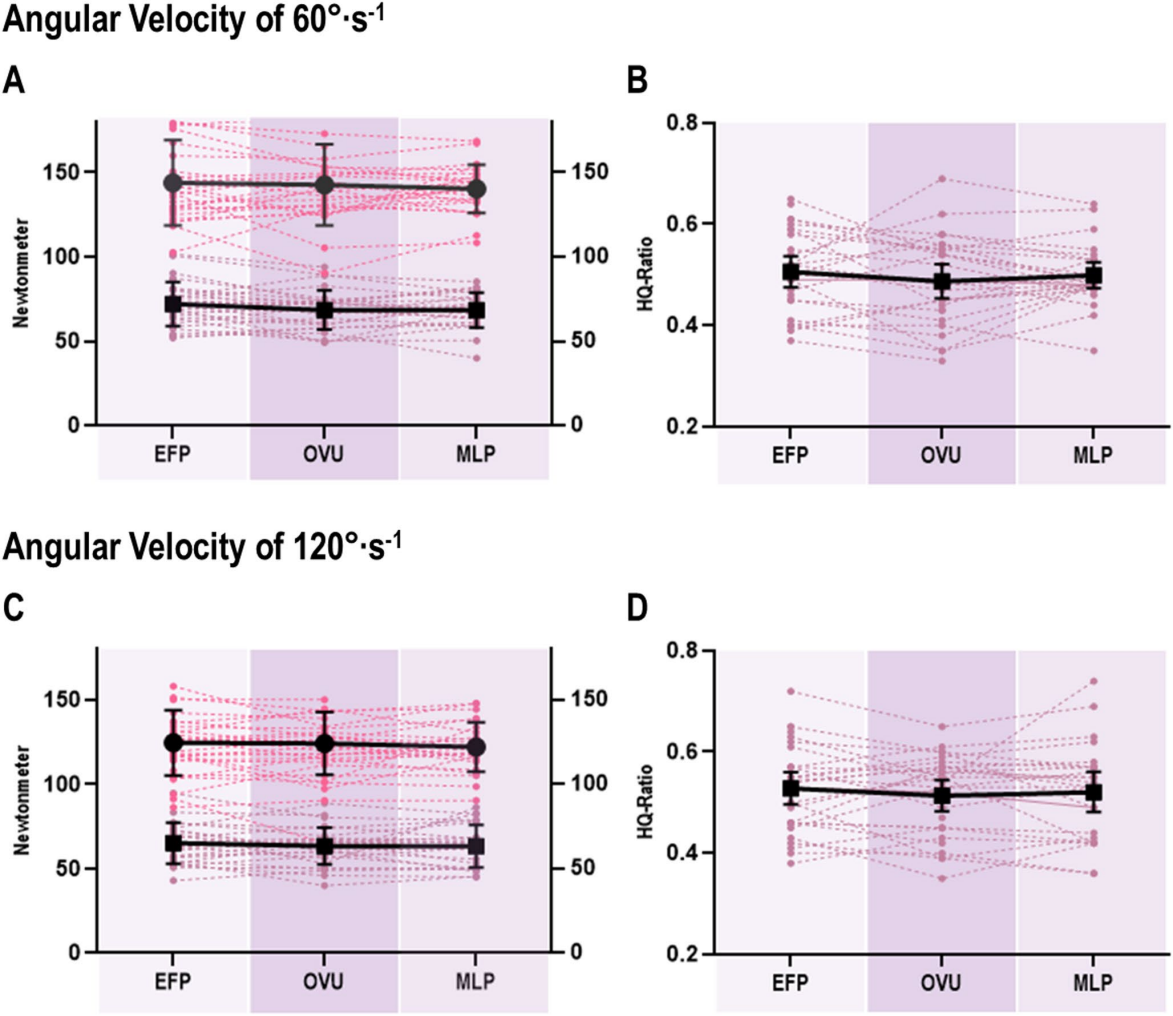
**A** Peak Torque values of knee extension (displayed as dots) and knee flexion (displayed as squares) at 60°∙s^− 1^ in Newtonmeter across the menstrual cycle. **B** Values of hamstring-to-quadriceps ratio across the menstrual cycle at 60°∙s^− 1^. **C** Peak Torque values of knee extension (displayed as dots) and knee flexion (displayed as squares) at 120°∙s^− 1^ in Newtonmeter across the menstrual cycle. **D** Values of hamstring-to-quadriceps ratio across the menstrual cycle at 120°∙s^− 1^. *EFP* early follicular phase, *OVU* ovulation, *MLP* mid-luteal phase

We tracked the menstrual cycle of participants two months prior to the measurements with a web application (*Athlete Monitoring™*, FITSTATS Technologies Inc., Moncton, Canada). To determine ovulation, participants were asked to detect the rise of luteinizing hormone (LH) by using urinary test strips at home (*DAVID One*, Runbio Biotech Co., Ltd., Shantou City, China), When the test strip turned positive following a surge of LH, participants were asked to attend the laboratory within 48 h. Seven to nine days later, the measurement in mid-luteal phase was conducted Fig. 1.

Measurements were conducted on cycle day 2.6 (± 1.2) in early follicular phase, day 15.3 (± 2.3) at ovulation, and day 22.1 (± 4.0) in mid-luteal phase. Mean cycle length was 28.2 days (± 2.9).

### Measurement outcomes

Primary outcome was conventional hamstring-to-quadriceps ratio (HQ Ratio) between isokinetic concentric peak torque of leg flexion and isokinetic concentric peak torque of leg extension in angular velocities of (1) 60°∙s^− 1^ and (2) 120°∙s^− 1^. Secondary outcomes were concentric isokinetic peak torque values of leg extension and leg flexion in angular velocities of (1) 60°∙s^− 1^ and (2) 120°∙s^− 1^.

While 60°·s⁻¹ is the standard angular velocity for isokinetic strength testing, peak torque was additionally assessed at 120°·s⁻¹, as previous research has demonstrated higher reliability of the unilateral hamstring-to-quadriceps ratio at this velocity compared to 180°·s⁻¹ (Impellizzeri et al. 2008).

We further chose to assess the conventional HQ ratio, as the functional ratio would have required eccentric hamstring contractions, which may induce muscle damage and subsequently elicit a repeated bout effect (Coratella and Bertinato 2015). Given that participants were assessed at multiple time points within a maximum interval of 15 days, we aimed to minimize potential confounding effects related to muscle damage and subsequent adaptive responses.

### Experimental procedures

Isokinetic strength measurements were conducted with the dynamometer Isomed 2000 (D. & R. Ferstl GmbH, Hernau, Germany). Participants were placed on the device in a seated position with a hip angle of 80° and fixed with straps to minimize movements. The fulcrum of the lever arm was aligned with the lateral femoral epicondyle of the participant’s dominant leg to assure efficient force transmission. To determine leg dominance, participants were asked, with which leg they preferably kick a football. After a standardized warm-up protocol and embedded in further measurements, peak torque values of knee extension and flexion were assessed within five consecutive concentric repetitions. We recorded highest values of extension and flexion in Newtonmeter (Nm) for subsequent data analysis.

### Data analysis and visualization

An a priori power analysis was conducted using G*Power (version 3.1.9.7) for a repeated-measures ANOVA within subjects. The analysis assumed a medium effect size of *f* = 0.25 (equivalent to η² = 0.06), an α level of 0.05, and power (1 – β) of 0.80. The model specified one group with three repeated measurements, a nonsphericity correction of ε = 1, and a correlation among repeated measures of 0.50. The analysis indicated that a total sample size of *N* = 28 would be required to detect the specified effect, yielding a nominal actual power of 0.81.

Data management was performed using *Microsoft*^®^ *Excel*^®^ 2019 (Version 18.08, Build 10416.20058, Microsoft Corporation, Redmond) and data analysis was performed with *RStudio* (*R version* 4.5.0; “*How about a Twenty-Six*”, RStudio 2025.09.0, Boston, MA.) using the *lme4* package for linear mixed models. We included a random intercept for participant ID to account for interindividual variability. Measurement timepoints were modelled as factors. The final model was specified as: Variable ~ Measurement Timepoint + (1 | Participant ID). Model performance was evaluated using marginal $\:{R}^{2}$ (variance explained by fixed effects), conditional R^2^ (variance explained by both fixed and random effects), and the intraclass correlation coefficient (ICC) as a measure of between-subject variance. Pairwise comparisons were conducted using estimated marginal means, and standardized effect sizes (Cohen’s d) were calculated. Data was visualized using *GraphPad Prism*, Version 10.1 (*GraphPad Software*, Boston MA, USA).

## Results

### Measurement outcomes

Peak torque values of knee extension and flexion and conventional hamstring-to-quadriceps ratios at 60°∙s^− 1^ and 120°∙s^− 1^ across cycle phases are displayed in Table 1.

**Table 1:** Peak Torque Values of Knee Extension, Knee Flexion and hamstring-to-quadriceps ratio (HQ ratio) in two different angular velocities across early follicular phase (EFP), ovulation (OVU), and mid-luteal phase (MLP) displayed as mean values and standard deviation (SD) and 95% confidence interval (95% CI)

Variable	Early Follicular Phase			Ovulation			Mid-Luteal Phase		
	Mean (SD)	95% CI		Mean (SD)	95% CI		Mean (SD)	95% CI	
*Angular Velocity of 60°∙s-1*									
Knee Extenion (Nm)	143.93 (25.30)	[134.31, 153.56]		142.76 (24.03)	[133.44, 152.08]		140.27 (14.28)	[134.37, 146.16]	
Knee Flexion (Nm)	72.10 (13.04)	[67.14, 77.06]		68.50 (11.56)	[64.02, 72.98]		68.39 (10.35)	[64.12, 72.66]	
HQ-Ratio	0.51 (0.08)	[0.47, 0.54]		0.49 (0.09)	[0.45, 0.52]		0.50 (0.06)	[0.47, 0.52]	
*Angular Velocity of 120°∙s-1*									
Knee Extension (Nm)	124.62 (19.46)	[117.35, 131.89]		124.14 (18.65)	[116.91, 131.38]		122.08 (14.63)	[116.04, 128.12]	
Knee Flexion (Nm)	65.21 (12.16)	[60.67, 69.75]		63.33 (11.02)	[59.06, 67.61]		63.23 (12.57)	[58.04, 68.42]	
HQ-Ratio	0.53 (0.09)	[0.50, 0.56]		0.51 (0.08)	[0.48, 0.54]		0.52 (0.10)	[0.48, 0.56]	

### Angular velocity of 60°∙s^− 1^

#### HQ Ratio

Analysis did not show differences of HQ ratio at 60°∙s^− 1^ across the menstrual cycle (F (2,5) = 0.86, *p* = 0.43). The intraclass correlation coefficient showed substantial between-participant variability (ICC = 0.65). The model yielded a marginal R^2^ of 0.008, implying that the fixed effects explained 0.8% of the variance. The conditional R^2^ was 0.73, indicating that the inclusion of random effects increased explained variance to 73%. Standardized mean differences were small between early follicular phase and ovulation (d = 0.34, 95% CI [−0.2; 0.89]), and early follicular and mid-luteal phase (d = 0.25, 95% CI [−0.31; 0.82]); trivial effect sizes were observed between ovulation and mid-luteal phase (d = −0.09, 95% CI [−0.65; 0.47]).

Isokinetic Peak Torque.

Analysis did not show differences in isokinetic peak torque of knee extension at 60°∙s^− 1^ across the menstrual cycle (F (2,50) = 0.5, *p* = 0.58). The intraclass correlation coefficient showed substantial between-participant variability (ICC = 0.82). The model yielded a marginal R^2^ of 0.002, implying that the fixed effects explained 0.2% of the variance. The conditional R^2^ was 0.82, indicating that the inclusion of random effects increased explained variance to 82%. Standardized mean differences were small between early follicular phase and ovulation (d = 0.21, 95% CI [−0.34; 0.76]), and trivial between early follicular phase and mid-luteal phase (d = −0.06, 95% CI [−0.63; 0.51]), and ovulation and mid-luteal phase (d = −0.27, 95% CI [−0.84; 0.29]).

Analysis did not show differences in isokinetic peak torque of knee flexion at 60°∙s^− 1^ across the menstrual cycle (F (2,50 = 2.43, *p* = 0.1). The intraclass correlation coefficient showed substantial between-participant variability (ICC = 0.71). The model yielded a marginal R^2^ of 0.02, implying that the fixed effects explained 0.2% of the variance. The conditional R^2^ was 0.71, indicating that the inclusion of random effects increased explained variance to 71%. Standardized mean differences were moderate between early follicular phase and ovulation (d = 0.57, 95% CI [−0.02; 1.12]), and early follicular phase and mid-luteal phase (d = 0.05, 95% CI [−0.13; 1.02]) and small between ovulation and mid-luteal phase (d = −0.12, 95% CI [−0.68; 0.44]).

### Angular velocity of 120°∙s^− 1^

#### HQ Ratio

Analysis did not show differences of HQ ratio at 120°∙s^− 1^ across menstrual cycle phases (F (2, 52) = 0.46, *p* = 0.64). The intraclass correlation coefficient showed substantial between-participant variability (ICC = 0.72) The model yielded a marginal R^2^ of 0.003, implying that the fixed effects explained 0.3% of the variance. The conditional R^2^ was 0.72, indicating that the inclusion of random effects increased explained variance to 72%. Standardized mean differences were small between early follicular phase and ovulation (d = 0.25, 95% CI [−0.29; 0.79]), and between early follicular phase and mid-luteal phase (d = 0.17, 95% CI [−0.39; 0.73]), and trivial between ovulation and mid-luteal phase (d = −0.08, 95% CI [−0.64; 0.48]).

Isokinetic Peak Torque.

Analysis did not show differences in isokinetic peak torque of knee extension at 120°∙s^− 1^ across the menstrual cycle (F (2,51) = 0.29, *p* = 0.75). The intraclass correlation coefficient showed substantial between-participant variability (ICC = 0.84). The model yielded a marginal R^2^ of 0.001, implying that the fixed effects explained 0.1% of the variance. The conditional R^2^ was 0.85, indicating that the inclusion of random effects increased explained variance to 85%. Standardized mean differences were small between early follicular phase and ovulation (d = 0.16, 95% CI [−0.38; 0.70]), and early follicular and mid-luteal phase (d = 0.19, 95% CI [−0.76; 0.37]), and trivial between early follicular phase and ovulation (d = −0.03, 95% CI [−0.6; 0.53]).

Analysis did not show in isokinetic peak torque of knee flexion at 120°∙s^− 1^ across the menstrual cycle (F (2,52) = 0.48, *p* = 0.62). The intraclass correlation coefficient showed substantial between-participant variability (ICC = 0.65). The model yielded a marginal R^2^ of 0.004, implying that the fixed effects explained 0.4% of the variance. The conditional R^2^ was 0.65, indicating that the inclusion of random effects increased explained variance to 65%. Standardized mean differences were small between each comparison: early follicular phase and ovulation (d = 0.26, 95% CI [−0.28; 0.8]), early follicular phase and mid-luteal phase (d = 0.17, 95% CI [−0.43; 0.68]), and ovulation and mid-luteal phase (d = −0.14, 95% CI [−0.7; 0.43]).

## Discussion

This study aimed to evaluate differences in conventional hamstring-to-quadriceps ratio across the menstrual cycle at two different angular velocities. It has been shown that knee laxity may vary across the menstrual cycle and may increase simultaneously with peaking estradiol concentration (Somerson et al. 2019, Herzberg et al. 2017, Hewett et al. 2007). Strength ratios between leg extensors and flexors due to altered neuromuscular activation patterns across the cycle could be one reason for this outline observed and hence, risk factors for injuries of knee-adjacent ligaments may be increased during those phases (Möck et al. 2023, Korpinen et al. 2025).

### Does hamstring to quadriceps ratio vary across the menstrual cycle?

The results of this study did not report differences of hamstring-to-quadriceps ratio either at 60°∙s^− 1^, nor at 120°∙s^− 1^ angular velocity across the assessed cycle phases. Confidence intervals were narrow at each measurement time point, indicating low variability and high precision of the estimates, with no meaningful effect of cycle phases on the conventional HQ ratio. Furthermore, phase-wise comparisons revealed only trivial-to-small effect sizes for HQ ratio at both angular velocities, indicating no clinically meaningful variation across menstrual cycle phases.

Isokinetic peak torque values of leg flexors and extensors followed the same course across the menstrual cycle and did not differ significantly between the evaluated phases. Our findings suggest that the capacity to generate force in both muscle groups remains stable across the assessed menstrual cycle phases, irrespective of potential hormonal fluctuations. However, since we did not evaluate hormone concentrations in blood or saliva, these results are only valid for assessed timepoints in measured cycle phases. It may still be possible that hormone concentrations varied within these cycle phases and hence, an association between hormone concentrations and HQ ratio cannot be drawn based on the findings of this study design.

Although menstrual cycle phases have been linked to neuromuscular modulation (e.g., motor unit recruitment, tendon stiffness) (Tenan et al. 2013, Ansdell et al. 2019), evidence suggests that maximal voluntary strength under controlled isokinetic conditions is relatively robust over the course of the menstrual cycle (Blagrove et al. 2020).

These results align with Pournasiri et al. (2023) who reported no significant differences of HQ ratio across the menstrual cycle; however, they applied the functional hamstring-to-quadriceps ratio, dividing eccentric peak torque of hamstrings by concentric peak torque of the quadriceps. Hence, results are only comparable to a limited extent. However, Pournasiri et al. (2023) reported that isokinetic and isometric peak torque values at 60°∙s^− 1^ differed significantly between menstrual cycle phases, indicating higher strength values at ovulation compared to early follicular and mid-luteal phase (Pournasiri et al. 2023), contrasting the course of strength values of our study.

### Neuromuscular activation patterns across the menstrual cyle

To evaluate neuromuscular activation patterns across the menstrual cycle, Khowailed et al. (2015) investigated electromyographic activity of hamstrings and quadriceps during running (Khowailed et al. 2015). Their findings indicated greater quadriceps activity in early follicular phase, whereas hamstring muscle activity was increased during ovulation (Khowailed et al. 2015). In addition, the ratio of hamstrings-to-quadriceps activity differed significantly between early follicular and ovulatory phase. The authors concluded that neuromuscular control patterns may alter in response to estradiol concentrations (Khowailed et al. 2015).

However, as this study investigated activation patterns during running, it remains unclear whether similar alterations occur during a strength-dominant tasks, or during sport-specific movements, such as cutting and landing (Hewett et al. 2008). Consistent with these findings, other studies have also reported changes in neuromuscular activation patterns across the menstrual cycle (Ansdell et al. 2019, Tenan et al. 2013). Tenan et al. (2013) demonstrated that initial firing rate of the vastus medialis and vastus medialis oblique varied between cycle phase, with significant differences observed between muscle at ovulation and during the mid- and late luteal phase (Tenan et al. 2013). Similarly, Ansdell et al. (2019) reported that neuromuscular function, assessed via and motor nerve stimulation and transcranial magnetic stimulation, was highest at day 14 of the menstrual cycle compared to day 2 and 21.

Taken together, these results suggest that neuromuscular activation patterns may vary across menstrual cycle phases. However, most studies report stable strength outcomes across cycle phases (Blagrove et al. 2020). Therefore, it may be assumed that cycle-related alterations in neuromuscular activation have only a limited impact on concentric strength performance.

### Hamstring to quadriceps ratio in a female population

Our results demonstrated HQ ratios varying from 0.49 (± 0.09) at ovulation to 0.51 (± 0.08) in early follicular phase at 60°∙s^− 1^ and 0.51 (± 0.08) at ovulation to 0.53 (± 0.09) in early follicular phase at 120°∙s^− 1^ angular velocity. Korpinen et al. (2025) reported conventional HQ ratio values of 0.56 (± 0.08) in team sports, 0.57 (± 0.08) in field sports, and 0.56 (± 0.08) in court sports for a female population, that are marginally higher than values from our study. Hewett et al. (2008) showed gender differences in HQ ratio at each velocity, and significant differences between males and females at movement velocities of 120°∙s^− 1^ and higher.

Several studies associated values below 0.6 with a higher incidence of knee injuries (Mandroukas et al. 2023, Lang et al. 2017); however these values are derived from research with male athletes (Korpinen et al. 2025). Korpinen et al. (2025) showed that cut off values were not met neither for female team sport athletes, nor field or court athletes (Korpinen et al. 2025). Those results indicate that HQ Ratio seems to be generally higher in males than in females, questioning the validity of using identical cutoff values for both sexes. Consequently, cut off values in female athletes need to be established based on gender specific data before HQ ratio can be applied as possible predictor for injury risk in a female population.

### Hamstring to quadriceps ratio as predictor for ACL injury risk

Impaired hamstring strength capacity of female athletes could be a reason for gender differences in HQ ratio and thus higher injury risk (Hewett et al. 2008). Hamstrings act as dynamic stabilizers of the knee joint and contribute to ACL protection by limiting anterior tibial translation. Hence, hamstring strength, but also muscular fatigue resistance are connected to a reduced risk of non-contact ACL ruptures (Franco et al. 2024). Holcomb et al. (2007) investigated the effect of a 6-week strength training program emphasizing two hamstring specific exercises on conventional and functional HQ ratios in female soccer players (Holcomb et al. 2007). Results showed that the program indicated a significant effect on functional, but not on conventional HQ ratio. Especially in landing and cutting movements, the eccentric strength capacity of hamstrings may be a factor in stabilizing the knee joint. Hence, functional HQ ratio reflects greater proximity to real life situations. Myer et al. (2009) reported that females who suffered an ACL injury displayed reduced hamstring strength compared to uninjured males (Myer et al. 2009). However, they examined the conventional hamstring ratio at 300°·s⁻¹; therefore, it remains unclear whether the functional HQ ratio at other velocities may reflect distinct risk factors more precisely. Nevertheless, it should be acknowledged that anterior cruciate ligament (ACL) injuries are multifactorial in nature, and neither the HQ ratio nor hamstring strength alone can be considered the sole determinant of increased injury risk (Kolodziej et al. 2023, Kolodziej et al. 2021). Högberg et al. (2024) investigated the association between HQ ratio and second ACL injuries in women with restructured ACL. They showed that HQ ratio was not significantly associated with the odds of re-injury when accounting for multiple factors, such as time from reconstruction to preinjury sport level, graft choice, age, body mass index, and preinjury activity. Hence, it may be stated that the validity of HQ ratio to predict injuries and reinjury is limited.

### Practical implications

The present findings indicate that the conventional hamstring-to-quadriceps ratio at 60°·s⁻¹ and 120°·s⁻¹ remains stable across menstrual cycle phases in eumenorrheic women. Consequently, cycle phase–adapted scheduling of isokinetic testing appears unnecessary when evaluating the conventional HQ ratio. However, given evidence suggesting cycle-related alterations in neuromuscular activation patterns and the limited predictive validity of the conventional HQ ratio for ACL injury risk, and furthermore, a lack of data in regard of female athletes, practitioners should avoid relying on this parameter in isolation. Instead, emphasis should be placed on eccentric hamstring strength, neuromuscular control, and sport-specific movement training, particularly in tasks involving cutting and landing. Importantly, the present findings contribute to the ongoing debate regarding menstrual cycle effects on neuromuscular performance. While many studies focus on potential performance fluctuations across the cycle, evidence demonstrating stability of strength-related parameters is equally important for practitioners and researchers. Our results suggest that conventional HQ ratio may represent a relatively stable parameter that is not substantially influenced by menstrual cycle phase. However, given the modest sample size and methodological characteristics, the generalizability of these findings may be limited to the present study design and population.

### Limitations

Although we conducted the study with great methodological rigor, certain limitations must be considered when interpreting the present results.

We only evaluated the conventional ratio considering concentric peak torque of hamstrings and quadriceps; however, functional ratio could have a greater validity regarding strength imbalances in real life situations such as landing, pivoting or cutting.

Furthermore, we did not measure knee laxity or electromyographic activity in leg flexors and extensors. Thus, we cannot make statements about knee laxity or muscle activation patterns in the assessed cohort. While we tracked the menstrual cycle and detected ovulation by rise of LH in urine, hormone concentrations were not assessed either in saliva or serum. Hence, associations between hormone concentrations and HQ ratio cannot be made. Also, measurements were conducted in a fixed sequence from the early follicular phase through ovulation to the mid-luteal phase, potentially amplifying learning effects. However, since values did not improve across the phases, a systematic learning effect appears unlikely.

## Conclusion

This study does not provide evidence that the conventional HQ ratio of the dominant leg differs between menstrual cycle phases in eumenorrheic females at different angular velocities. Furthermore, concentric peak torque values of leg extension and flexion remained constant across the cycle, opposing a possible influence of cycle phases on strength outcomes. From a clinical perspective, these findings suggest that isokinetic testing of conventional HQ ratio does not need to be scheduled according to menstrual cycle phases. However, future research should investigate the functional HQ ratio across menstrual cycle phases, as eccentric hamstring contractions more closely reflect real-life movement demands.

## Data Availability

Data is available on request.
